# Systemic inflammation score as a preoperative prognostic factor for patients with pT2–T4 resectable gastric cancer: a retrospective study

**DOI:** 10.1186/s12893-023-01904-z

**Published:** 2023-01-12

**Authors:** Takuro Matsumoto, Shinji Ohki, Akinao Kaneta, Akira Matsuishi, Yuya Maruyama, Leo Yamada, Takeshi Tada, Hiroyuki Hanayama, Yohei Watanabe, Suguru Hayase, Hirokazu Okayama, Wataru Sakamoto, Tomoyuki Momma, Zenichiro Saze, Koji Kono

**Affiliations:** 1 Department of Gastrointestinal Tract Surgery, Fukushima Medical University, 1 Hikarigaoka, Fukushima, 960-1295 Japan; 2 Shirakawa Kosei General Hospital, 2-1 Toyochikamiyajirou, Shirakawa, Fukushima 961-005 Japan

**Keywords:** Gastric cancer, Prognostic factor, Systemic inflammation score

## Abstract

**Background:**

Systemic inflammation has been reported to be associated with cancer progression and metastasis. Systemic inflammation score (SIS), calculated from preoperative serum albumin level and lymphocyte-to-monocyte ratio, has been shown to be a novel prognostic factor for several types of tumors. This study aimed to evaluate the prognostic value of the SIS in patients with pT2–4 resectable gastric cancer (GC).

**Methods:**

Total 97 patients with pT2–4 GC who underwent curative surgery from 322 cases between 2009 and 2015 in Fukushima Medical University Hospital were included. We performed univariate and multivariate analyses to evaluate the usefulness of preoperative SIS and other prognostic factors for relapse-free survival (RFS) and overall survival (OS).

**Results:**

The higher SIS score was associated with undifferentiated cancer and recurrence. Univariate analysis of RFS identified deeper tumor invasion and higher SIS were significant risk factors and multivariate analysis revealed that both of them were independent prognostic factors for RFS. As for OS, age, tumor invasion, SIS and LNR were significantly correlated with RFS. In multivariate analysis, tumor invasion, SIS and LNR were independent prognostic factors for OS.

**Conclusions:**

SIS was an independent prognostic factor for RFS and OS in pT2–4 resectable gastric cancer patients who underwent curative gastrectomy.

**Supplementary Information:**

The online version contains supplementary material available at 10.1186/s12893-023-01904-z.

## Background

Gastric cancer (GC) is the third most common cause of cancer death worldwide [1]. Despite significant improvements in the therapies for GC patients, the survival of patients with advanced GC still remains poor [2, 3]. By far, several prognostic models have been used to predict prognosis for GC patients. However accurate prediction of individual survival remains controversial.

In addition to local inflammatory reaction, cancer patients often exhibit systemic inflammatory response, such as changes in peripheral blood cell counts and C-reactive protein (CRP), decreased hemoglobin and serum albumin (Alb) levels [4, 5]. Systemic inflammation has been reported to play a critical role in cancer progression and metastasis, and its interactions with host-tumor are currently recognized as the seventh hallmark of cancer [6–9]. Accordingly, prognostic factors based on the several ratios of the circulating blood cells, such as neutrophil-to-lymphocyte ratio (NLR) [10], lymphocyte-to-monocyte ratio (LMR) [11], Lymph node ratio (LNR) [12, 13], CRP-to-albumin ratio (CAR) [14, 15], modified Glasgow Prognostic Score (mGPS) [16, 17], have been developed and reported to be associated with poor survival in cancer patients.

Systemic inflammation score (SIS), based on the preoperative serum albumin level and LMR, was reported to be a prognostic marker for clear cell renal cell carcinoma (ccRCC) and colorectal cancer [18, 19]. Recently, some papers have reported on the association between SIS and poor prognosis in GC patients as well [20–23]. However, the prediction of GC prognosis by SIS is not yet common and further evidence needs to be accumulated.

The purpose of this study was to evaluate the clinical significance of preoperative SIS for relapse-free survival (RFS) and overall survival (OS) of GC patients with curative gastrectomy, compared to other prognostic biomarkers.

## Methods

### Patients

**Fig. 1:**
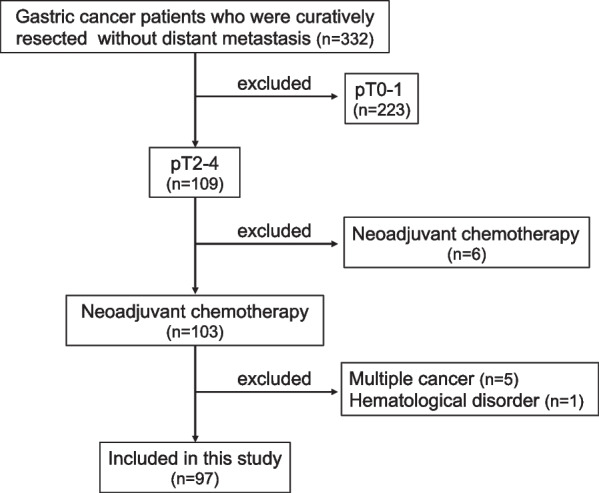
Flow chart of included and excluded criteria

This retrospective study recruited GC patients who were curatively resected at Fukushima Medical University Hospital, based on 322 cases between January 2009 and September 2015. The inclusion criteria were the following: (a) pT2–4 advanced GC according to the Japanese Classification of Gastric Carcinoma, the 15th Edition [24] and (b) curatively resected with systemic lymphadenectomy. The exclusion criteria were the following: (a) neoadjuvant chemotherapy, (b) distant metastasis, (c) multiple cancer and (d) hematological disorder. Finally, 97 GC patients were included in this study (Fig. 1). All clinical data were retrospectively collected from medical records.

### Definition of prognostic markers

The patients’ blood tests were performed before surgery. These included the lymphocyte count, monocyte count, platelet count, serum Alb and CRP levels.

LNR was defined as the ratio of positive divided by the total number of examined nodes [25]. mGPS was determined by serum Alb and CRP levels was defined based on the previously studies [17]. SIS was formulated according to serum Alb and LMR as follows: a score of 0 indicated patients with Alb ≥ 4.0 g/dL and LMR ≥ 4.44; a score of 1 indicated those with either Alb < 4.0 g/dL or LMR < 4.44; and a score of 2 indicated those with both Alb < 4.0 g/dL and LMR < 4.44 [18] (Table 1). CAR and LNR were divided into two groups by median, and the median (interquartile range) of each factor was 0.03 (0.01–0.09) and 0.067 (0.020–0.152). mGPS and SIS were also classified into two groups by scores.

**Table 1:** Definition of modified Glasgow Prognostic Score (mGPS) and Systemic Inflammation Score (SIS)

								Score
mGPS								
CRP		≤	1.0 mg/dL	And	Alb	≥	3.5 g/dL	0
CRP		>	1.0 mg/dL					1
CRP		>	1.0 mg/dL	And	Alb	<	3.5 g/dL	2
SIS								
LMR		≥	4.44	And	Alb	≥	4.0 g/dL	0
LMR		<	4.44	Or	Alb	<	4.0 g/dL	1
LMR		<	4.44	And	Alb	<	4.0 g/dL	2

### Follow-up investigation

All patients received postoperative follow-up every 3 months up to 2 years and every 6 months during 3–5 years after surgery and annually afterward. The routine follow-up included physical examinations, laboratory tests, enhanced CT and annual upper gastrointestinal endoscopy. Relapse free survival (RFS) and Overall survival (OS) were defined as the interval from the date of surgery to the date of recurrence and death from any cause, respectively.

### Statistical analysis

Fisher’s exact test was used to compare patient groups. Survival curves were plotted by the Kaplan–Meier method, and significance was determined by the log-rank test. Univariate and multivariate analysis were performed with the Cox proportional hazards model. ROC curves were used to evaluate their diagnostic ability.

A two-sided P-value less than 0.05 was considered to be statistically significant. All statistical analysis was performed using SPSS 26 (IBM Corporation) or GraphPad Prism v6.04 (Graphpad Software Inc.).

## Results

### Patient characteristics according to the SIS score

Totally, 97 GC patients were included in this study. The clinicopathological characteristics of the patients were shown in Table 2. There were 69 (71.1%) males and 28 (28.9%) females. The median age was 68.7 years (range 31–90 years). All parameters except patient’s age were divided into two groups. No significant differences between SIS 0–1 and SIS 2 were observed with respect to age, gender, tumor invasion, lymph node metastasis, lymphatic invasion, vascular invasion, administration of adjuvant therapy and surgical procedure. The high SIS score was significantly correlated with undifferentiated cancer and recurrence.

**Table 2:** Association between the systemic inflammation score (SIS) and clinicopathological findings

	Total (n = 97)	SIS		P value
		0–1 (n = 59)	2 (n = 38)	
Age	68.7 (± 12.6)	67.8 (± 12.6)	71.6 (± 12.6)	0.1358
Gender				0.8189
Male	69	41	28	
Female	28	18	10	
Histological type				0.0209*
Differentiated	43	32	11	
Undifferentiated	54	27	27	
pT				0.1444
T2,3	51	35	16	
T4	46	24	22	
pN				0.7897
−	19	13	6	
+	78	46	32	
Lymphatic invasion				> 0.9999
−	7	4	3	
+	90	55	35	
Venous invasion				0.4755
−	9	7	2	
+	88	52	36	
Adjuvant chemotherapy				0.5294
−	37	21	16	
+	60	38	22	
Operation				0.8354
DG + PG	44	26	18	
TG	53	33	20	
Recurrence				0.0207*
−	78	52	26	
+	19	7	12	

*P-value < 0.05 was considered to be statistically significant

*DG* distal gastrectomy, *PG* proximal gastrectomy, *TG* total gastrectomy

### ROC curve analyses for representative prognostic factors of cancer

ROC curve analyses were used to evaluate the significance of representative prognostic factors. Area under the curves (AUC) of the LMR [AUC 0.7095, 95% CI 0.5955–0.8235], that is a component of SIS, was highest in RFS (Additional file 1: Fig. S1). In OS, LNR [AUC 0.7394, 95% CI 0.6201–0.8587] had the highest AUC, but LMR [AUC 0.6989, 95% CI 0.5958–0.8020] was also close to it (Additional file 1: Fig. S2).

### Survival analysis for prognostic impact of SIS on RFS and OS

**Fig. 2:**
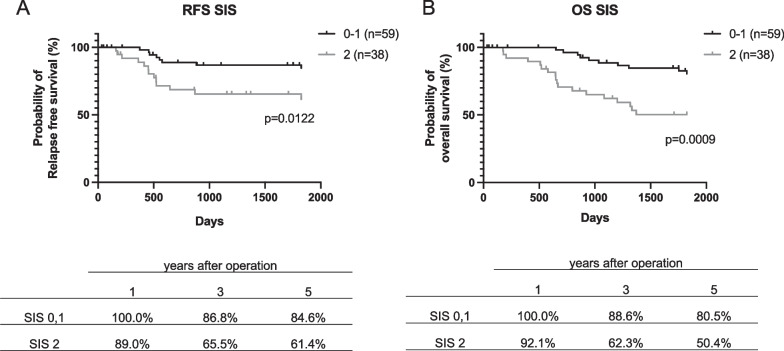
Kaplan–Meier analysis for relapse-free survival (**A**) and overall survival (**B**) according to SIS

The median follow-up time of patients was 1825 days (range 33–1825 days). From the Kaplan–Meier survival curve, higher SIS scores was significantly associated with poorer RFS and OS (Fig. 2). Table 3 shows the results of univariate and multivariate analyses for RFS. Univariate analysis demonstrated that tumor invasion and SIS score, but not other scores, were significantly correlated with RFS. Also, in multivariate analysis, tumor invasion [HR 3.162, 95% CI 1.212–8.251, p = 0.019] and SIS [HR 2.847, 95% CI 1.172–6.919, p = 0.021] were significantly associated with RFS. Regarding OS, age, tumor invasion, SIS score and LNR were associated with OS in univariate analysis. Multivariate analysis revealed a significant association between OS and age [HR 3.537, 95% CI 1.406–8.901, p = 0.007], tumor invasion [HR 4.396, 95% CI 1.820–10.622, p = 0.001], SIS score [HR 3.558, 95% CI 1.562–8.107, p = 0.003] and LNR score [HR 4.734, 95% CI 1.844–12.153, p = 0.001] (Table 4). Whereas, other prognostic scores, including mGPS and CAR, were not shown to be independent predictive factor for OS.

**Table 3:** Univariate and multivariate analysis of clinicopathologic variables in relation to RFS

	Univariate analysis		Multivariate analysis	
	HR (95% CI)	P	HR (95% CI)	P
Age		0.328		
< 70	Reference			
≤ 70	1.553 (0.643–3.753)			
Gender		0.974		
Male	Reference			
Female	0.985 (0.397–2.443)			
Histological type		0.986		
Differentiated	Reference			
Undifferentiated	1.008 (0.424–2.393)			
pT		0.022*		0.019*
T2,3	Reference		Reference	
T4	3.035 (1.176–7.829)		3.162 (1.212–8.251)	
pN		0.103		
−	Reference			
+	5.315 (0.713–39.627)			
Lymphatic invasion		0.399		
−	Reference			
+	–			
Venous invasion		0.418		
−	Reference			
+	2.295 (0.308–17.105)			
Operation		0.292		
DG + PG	Reference			
TG	1.628 (0.657–4.034)			
Adjuvant chemotherapy		0.682		
−	Reference			
+	1.219 (0.473–3.143)			
SIS		0.013*		0.021*
0,1	Reference		Reference	
2	3.069 (1.270–7.417)		2.847 (1.172–6.919)	
mGPS		0.478		0.690
0	Reference		Reference	
1,2	1.557 (0.458–5.290)		1.284 (0.376–4.387)	
CAR		0.852		0.904
< 0.03	Reference		Reference	
0.03 ≤	0.922 (0.391–2.171)		1.056 (0.436–2.554)	
LNR		0.050		0.066
< 0.067	Reference		Reference	
0.067 ≤	2.481 (0.999–6.164)		2.409 (0.945–6.142)	

*P-value < 0.05 was considered to be statistically significant

**Table 4:** Univariate and multivariate analysis of clinicopathologic variables in relation to OS

	Univariate analysis		Multivariate analysis	
	HR (95% CI)	P	HR (95% CI)	P
Age		**0.013**		**0.007**
< 70	Reference		Reference	
≤ 70	2.975 (1.256–7.047)		3.537 (1.406–8.901)	
Gender		0.193		
Male	Reference			
Female	0.547 (0.221–1.356)			
Histologic type		0.905		
Differentiated	Reference			
Undifferentiated	1.048 (0.486–2.258)			
pT		0.007*		0.001*
T2,3	Reference		Reference	
T4	3.281 (1.387–7.763)		4.396 (1.820–10.622)	
pN		0.236		
−	Reference			
+	2.066 (0.621–6.868)			
Lymphatic invasion		0.946		
−	Reference			
+	0.951 (0.225–4.018)			
Venous invasion		0.620		
−	Reference			
+	1.439 (0.341–6.079)			
Operation		0.509		
DG + PG	Reference			
TG	1.301 (0.596–2.842)			
Adjuvant chemotherapy		0.488		
−	Reference			
+	0.758 (0.347–1.657)			
SIS		0.001*		0.003*
0,1	Reference		Reference	
2	3.848 (1.725–8.582)		3.558 (1.562–8.107)	
mGPS		0.388		0.881
0	Reference		Reference	
1,2	1.597 (0.552–4.621)		0.919 (0.304–2.780)	
CAR		0.128		0.105
< 0.03	Reference		Reference	
0.03 ≤	1.834 (0.839–4.009)		1.981 (0.867–4.525)	
LNR		0.003*		0.001*
< 0.067	Reference		Reference	
0.067 ≤	3.697 (1.559–8.767)		4.734 (1.844–12.153)	

*P-value < 0.05 was considered to be statistically significant

## Discussion

We analyzed 97 patients with pT2–4 advanced GC who underwent curative surgery. Preoperative SIS was correlated with histologic type and recurrence. In addition, we demonstrated that relapse-free and overall survival were significantly poorer in higher SIS score group in multivariate analysis.

Many studies have shown that systemic inflammation and nutrition are related to the outcomes of cancer patients [6, 26]. The SIS, which is based on the combination of preoperative LMR and serum Alb, was reported to have prognostic value in several malignancies. Low LMR levels means low lymphocyte and high monocyte counts in the blood. Low lymphocyte counts indicate the suppressed immune surveillance and can lead to cell proliferation, invasion and metastasis of cancer [27], whereas, tumor-associated macrophages, differentiated from monocyte, had been reported to contribute the tumor invasion, metastasis and therapeutic resistance in cancer [28, 29]. Therefore, several studies had reported that preoperative LMR was correlated with prognosis in various cancers [11, 30]. Serum albumin level is not an indicator of nutritional status, but also systemic inflammation response [26]. Low serum albumin level had also been reported to be related with poor prognosis in many types of cancer [31, 32].

Recently, several studies reported the usefulness of SIS in gastric cancer. Sato et al. showed that SIS can predict the incidence of postoperative complications and survival in pT2–4 GC patients after gastrectomy [22]. Ma et al. retrospectively calculated preoperative SIS in all Stage GC patients, in which SIS can predict 5-year OS better than NLR and maintained the predictive accuracy superiority throughout the observation period [23]. According to the report by Chen et al., preoperative SIS exceeded both mGPS and lymphocyte C-reactive protein score (LCS) in predicting the survival of Stage I–IV GC patients [20]. In the present study, we examined SIS score along with mGPS, CAR and LNR and demonstrated the correlation between systemic inflammation and the survival of advanced GC patients who underwent curative gastrectomy. We indicated that SIS was more significant than the other factors, including mSIS, CAR and LNR, in RFS and was significant along with LNR in OS. To our knowledge, this is the first report to show that SIS has a statistically significant difference in both RFS and OS.

There are some strengths of SIS as a prognostic factor. First, as it can be assessed prior to surgery, it may help in choosing the treatment for the individual patients. The study of Shoka et al. indicated that preoperative SIS was a significant predictor of postoperative pneumonia in GC patients [33]. Consideration of the need for preoperative nutritional management and complication control may reduce perioperative complications and improve prognosis. Second, SIS can be assessed readily and repeatedly because it is based on peripheral blood samples. In perioperative analysis, Hara et al. reported that SIS at 1 month after surgery could predict the tumor recurrence and survival of patients with Stage II–III GC [21]. Further studies are needed because SIS might be a predictor of recurrence and prognosis even during postoperative follow-up.

The strategies for perioperative chemotherapy for gastric cancer vary from country to country and still controversial. According to the Japanese gastric cancer treatment guidelines, adjuvant chemotherapy is recommended for Stage II/III GC patients [34]. In terms of neoadjuvant chemotherapy, several clinical trials have been conducted in Japan, but the evidence is limited and does not lead to clear recommendations [34, 35]. The advantages of neoadjuvant chemotherapy include possibility to administer chemotherapy more intensively, but the disadvantages include the difficulty of accurate preoperative diagnosis, the possibility of becoming unresectability due to cancer growth during chemotherapy and increased postoperative complications. Adjuvant chemotherapy can be given after accurate diagnosis by histopathological findings, but there is a possibility that adjuvant chemotherapy cannot be performed due to deterioration of the general condition or complications after surgery. Although further studies are needed to determine the optimal treatment strategy, prognostic factors such as SIS might be important to determine the treatment strategy.

Our study has several limitations. First, it was a retrospective and single-center study. Thus, it may have been subject to selection bias. Second, the number of cases in this study was small that the ability to detect significant difference might be low. Third, the occurrence of perioperative complications was not examined and may have acted as a confounding factor.

## Conclusions

Our study showed that the preoperative SIS may be a significant prognostic factor for advanced GC.

## Supplementary Information

**Additional file 1: Figure S1.** ROC curves of prognostic factors for RFS. **Figure S2.** ROC curves of prognostic factors for OS.

## Data Availability

The datasets generated and analyzed in this study are not publicly available due to the protection of personal information of the patients, but they are available from the corresponding author on reasonable request.

## Abbreviations

SIS: Systemic inflammation score
GC: Gastric cancer
RFS: Relapse free survival
OS: Overall survival
CRP: C-reactive protein
Alb: Albumin
NLR: Neutrophil-to-lymphocyte ratio
LMR: Lymphocyte-to-monocyte ratio
LNR: Lymph node ratio
CAR: CRP-to-albumin ratio
mGPS: Modified Glasgow Prognostic Score
ccRCC: Clear cell renal cell carcinoma
LCS: Lymphocyte C-reactive protein score
