# Supplementary material for: Systemic inflammation score as a preoperative prognostic factor for patients with pT2–T4 resectable gastric cancer: a retrospective study
Source: BMC Surg. 2023 Jan 12;23:8. doi: 10.1186/s12893-023-01904-z (PMC9837917; doi:10.1186/s12893-023-01904-z)

Additional Figure S1. ROC curves of prognostic factors for RFS


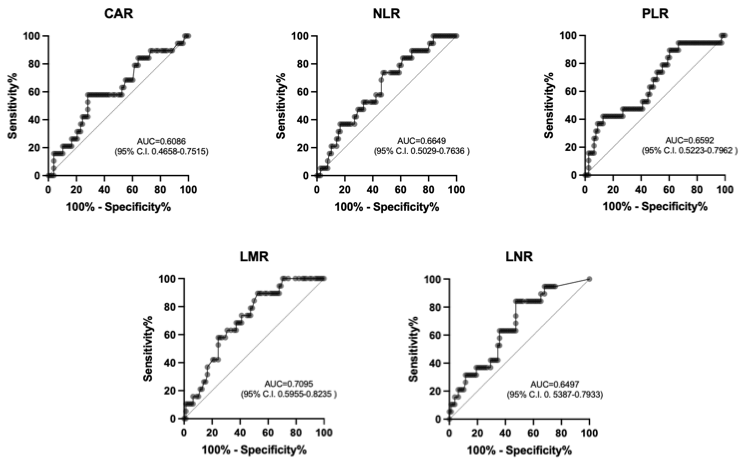


Additional Figure S2. ROC curves of prognostic factors for OS


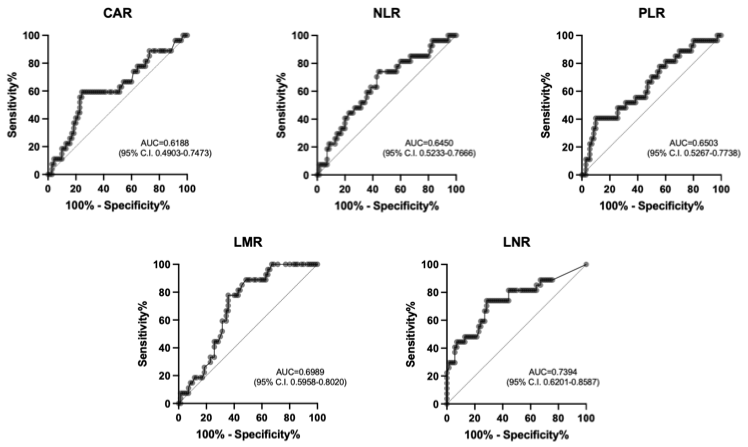

Supplement: Supplementary file 1 — Additional file 1: Figure S1. ROC curves of prognostic factors for RFS. Figure S2. ROC curves of prognostic factors for OS. [file 12893_2023_1904_MOESM1_ESM.docx]
